# The County Health Rankings: rationale and methods

**DOI:** 10.1186/s12963-015-0044-2

**Published:** 2015-04-17

**Authors:** Patrick L Remington, Bridget B Catlin, Keith P Gennuso

**Affiliations:** Department of Population Health Sciences, University of Wisconsin-Madison, 4263 Health Sciences Learning Center, 750 Highland Ave, Madison, WI 53705 USA; University of Wisconsin Population Health Institute, University of Wisconsin-Madison, 505 WARF Office Building, 610 Walnut St., Madison, WI 53726 USA; University of Wisconsin Population Health Institute, University of Wisconsin-Madison, 575C WARF Office Building, 610 Walnut St., Madison, WI 53726 USA

**Keywords:** Health rankings, Population health, Public health surveillance

## Abstract

**Background:**

Annually since 2010, the University of Wisconsin Population Health Institute and the Robert Wood Johnson Foundation have produced the *County Health Rankings*—a “population health checkup” for the nation’s over 3,000 counties. The purpose of this paper is to review the background and rationale for the *Rankings*, explain in detail the methods we use to create the health rankings in each state, and discuss the strengths and limitations associated with ranking the health of communities.

**Methods:**

We base the *Rankings* on a conceptual model of population health that includes both health outcomes (mortality and morbidity) and health factors (health behaviors, clinical care, social and economic factors, and the physical environment). Data for over 30 measures available at the county level are assembled from a number of national sources. Z-scores are calculated for each measure, multiplied by their assigned weights, and summed to create composite measure scores. Composite scores are then ordered and counties are ranked from best to worst health within each state.

**Results:**

Health outcomes and related health factors vary significantly within states, with over two-fold differences between the least healthy counties versus the healthiest counties for measures such as premature mortality, teen birth rates, and percent of children living in poverty. Ranking within each state depicts disparities that are not apparent when counties are ranked across the entire nation.

**Discussion:**

The *County Health Rankings* can be used to clearly demonstrate differences in health by place, raise awareness of the many factors that influence health, and stimulate community health improvement efforts. The *Rankings* draws upon the human instinct to compete by facilitating comparisons between neighboring or peer counties within states. Since no population health model, or rankings based off such models, will ever perfectly describe the health of its population, we encourage users to look to local sources of data to understand more about the health of their community.

## Introduction

Annually since 2010, the University of Wisconsin Population Health Institute and the Robert Wood Johnson Foundation have produced the *County Health Rankings*, a “population health checkup” for the nation’s over 3,000 counties (www.countyhealthrankings.org). The health of each county is ranked within each state – from the healthiest to the least healthy – using a model that summarizes the overall health outcomes of each county, as well as the factors that contribute to health [1]. The primary goal of the *Rankings* is to mobilize action toward community health by stimulating interest among the media and policymakers. The *Rankings*, in their current form, are an extension of the 2003 to 2008 annual Wisconsin County Health Rankings to the entire United States. In this paper, we review the background and rationale for the expanded *Rankings*, explain in detail the methods we use to create the health rankings in each state, and discuss the strengths and limitations associated with ranking the health of communities.

### Why do we rank?

Ranking is an effective, yet sometimes controversial approach to call attention to differences in a wide variety of areas in society—from the oft-cited *US News and World Report* rankings to international rankings of economy, education, or technology. Rankings, in general, are desirable because they have the ability to summarize complex information about a topic in a manner that is interpretable to everyone. Oliver suggests that population health rankings can be used to help set agendas — stimulating awareness, motivation, and debate over means to improved health outcomes, and to help establish broad responsibility for population health and the need for multisectorial collaboration to improve outcomes [2]. On the other hand, rankings are often criticized for a variety of reasons, including the arbitrariness of the measures used, inappropriate emphasis of insignificant differences, and the tendency of institutions to focus only on the elements included in the ranks [3,4]. Despite different views about the benefits of rankings, the public and media seems to have an insatiable appetite for them.

Population health rankings, such as the America’s Health Rankings and the *County Health Rankings*, are often used as a catalyst for the improvement of health by drawing attention to the areas that need improvement through an easily interpretable synthesis of objectively measured community health data [5]. Once the media and community leaders are made aware of problem areas, communities can be engaged to enact evidence-informed health policies and programs to improve health outcomes. Fundamentally, these health rankings are a tool to communicate with health professionals, local community leaders, and the general public, so that they make informed decisions about the health of their communities.

### Population health ranking history

The practice of population health ranking likely began as soon as health statistics began to be collected, compiled, and reported publicly. Since the 1960s, the CDC’s *Morbidity and Mortality Weekly Report* (MMWR) has reported health statistics for the leading causes of death and disability, often by geographic regions like states and metropolitan areas [6]. For example, in 1987, an MMWR publication ranked the rates of health risk behaviors by state, showing that the prevalence of overweight and smoking varied almost two-fold and alcohol-related behaviors varied up to six-fold by state [7]. This report led to a front-page story in the *Atlanta Journal-Constitution* that showed state ranks for each risk factor [8] followed by media interviews from around the nation. In 1988, one of the authors (PLR) published an article in the MMWR that ranked state-specific death rates due to ischemic heart disease [9], leading to an Associate Press headline that stated, “Midwest, Northeast city life hard on hearts” [10]. This report and the subsequent media attention led to calls to the CDC from health officials and legislators from the states with the highest death rates, insisting that the CDC refrain from publishing rankings in the MMWR.

In 1990, Northwestern National Life Insurance Company sponsored the publication of a report that summarized the health of the 50 states in the US and ranked them from healthiest to least healthy. These reports were unique as they measured the overall health of an entire state. The reports garnered attention in the media, leading to discussions about why health varied dramatically from one state to another [5]. Following this positive experience, Arundel Street Consulting recruited and conducted a Delphi panel that developed a method to compare the healthiness of the general population of each state with other states [11]. This report has been published annually since 1990, now produced by the United Health Foundation as “America’s Health Rankings”.

### *County Health Rankings* history

Based on the media interest from health rankings published in the MMWR and with the state health rankings, county health rankings were first proposed in 1994 by one of the authors (PLR), when he was a Chief Medical Officer at the Wisconsin Division of Public Health. However, efforts to produce these rankings through the state health department were not successful, due in part to concerns about potential backlash from local and statewide policy makers. A comment by a reviewer of an unsuccessful grant proposal submitted to the CDC stated that the release of the rankings “may be quite counterproductive. These often incite great resistance”.

In 2002, the Population Health Institute was established at the University of Wisconsin, with the mission of translating research into policy and practice. One of the first efforts of the Institute was to develop county health rankings for Wisconsin using a model similar to the model used to rank the health of states [12]. Our first report was released that year at a conference of Wisconsin local public health officials and included a press release for the local media. Although the use of ranks was considered, the report instead used a modification of the *Consumer Reports*-style circles to characterize quartiles from healthiest (Q1) to least healthy (Q4) [13]. This method was found to be difficult to interpret with little interest shown by the local media in reporting the results. Because of this experience, the report was revised and the quartiles were replaced with standard ranking from healthiest (#1) to least healthy (#72). Although this report used the same data as the earlier report, the use of ranking resulted in significant interest among the media and, as a result, more engagement of local health officials [14]. This report became the first in an annual series of “Wisconsin County Health Rankings” published annually thereafter through 2008.

We conducted an evaluation following the release of the 2006 Wisconsin County Health Rankings by searching newspaper, television, and radio coverage and by surveying local public health officials throughout the state [15]. More than 15 newspapers across the state covered the Rankings with headlines such as “Dane County’s residents among state’s healthiest;” “Rock County up, slightly, in health rankings;” “Florence County the healthiest in the state;” and “Washington county ranks 7^th^ healthiest county in state.” Newspaper articles often focused on specific strengths and weaknesses of their local area, such as: “Wealthy and healthy: Waukesha County fares well in new survey;” “Report: County fitter but smoking, drinking too much;” and “County’s health stats in decline: Poor air, smoking, lack of diplomas cause concern”. Nearly all (94%) of the 52 county health officers and regional epidemiologists who responded to our survey reported using the Wisconsin County Health Rankings in their work, primarily for educating policymakers and community partners, performing needs assessments, and identifying program targets.

During the six years that we produced the Wisconsin County Health Rankings, we were contacted by public health institutes in other states interested in using our model to rank counties in their state. Reports published in Tennessee, Kansas, and New Mexico received similar attention among policymakers and the media in each state [16,17]. Following a presentation about the Wisconsin County Health Rankings at a national public health conference [18], we began discussions with the Robert Wood Johnson Foundation about using our methods to rank every county in every state in the nation. In late 2008, the University of Wisconsin Population Health Institute received a grant from the Robert Wood Johnson Foundation to use the model and experience from the Wisconsin County Health Rankings to develop reports for counties in each of the 50 states. This project, entitled “Mobilizing Action Toward Community Health”, supported the development of the current *Rankings* for all 50 states.

In 2009, we convened a panel of national experts on population health and commissioned a series of publications examining population health metrics and incentives and partnerships for improvement [19-21]. Specifically, these publications examined measures in the five domains used in the Wisconsin County Health Rankings: health outcomes [22], health behaviors [23], health care [24], socioeconomic status [25], and environmental health [26]. Based on these reviews, we sought existing national data sources to determine availability and cost of data at the county level for each of the nation’s more than 3,000 counties.

## Methods

### The *County Health Rankings* model

**Figure 1:**
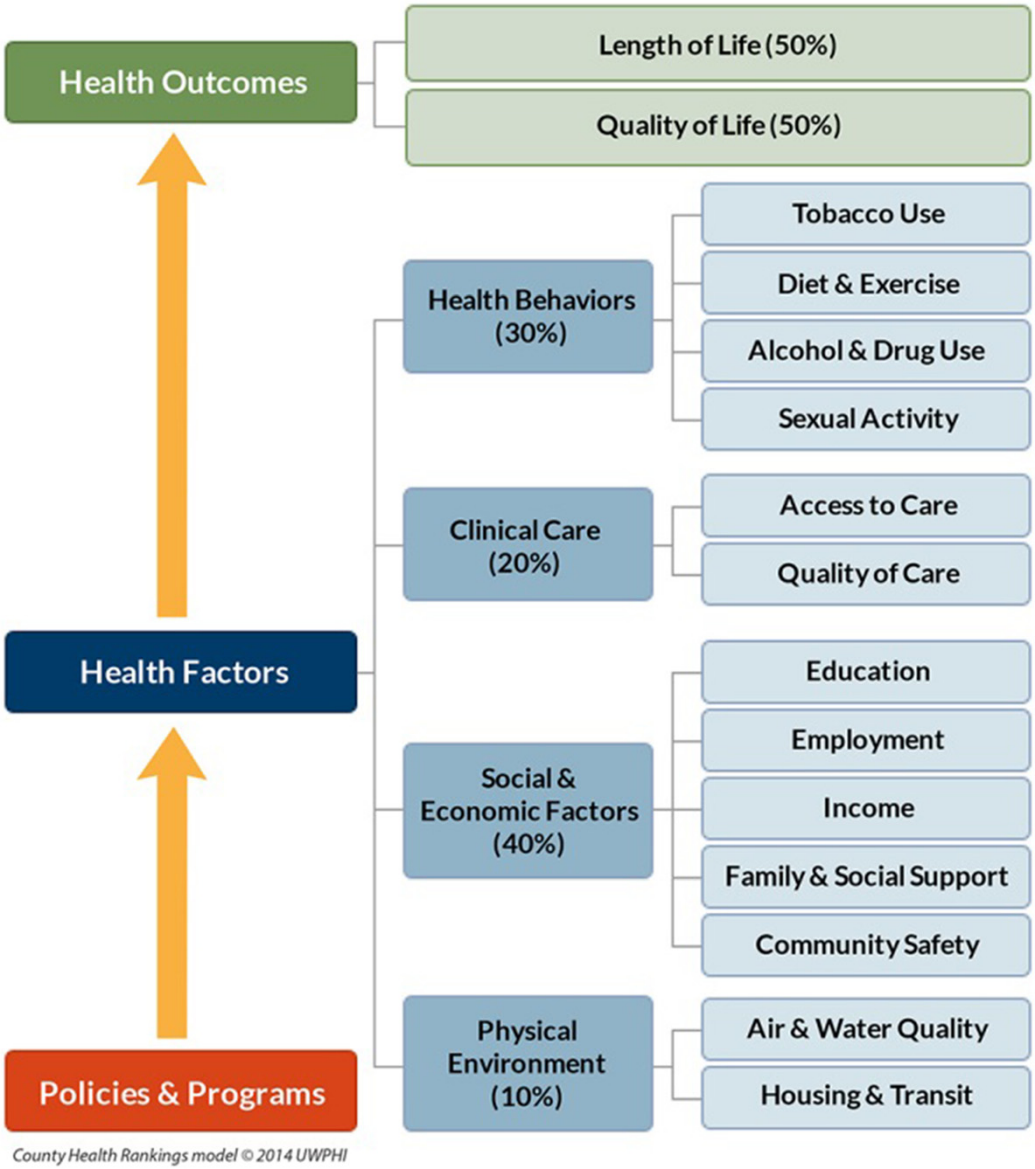
County Health Rankings Model.

We base the *Rankings* on a conceptual model (Figure 1) of population health that includes both health outcomes and health factors. Health outcomes reflect the current state of health in a county and are split broadly into two components; length of life and quality of life. Health factors are divided amongst four components thought to be modifiable determinants of the future health of a county. They include health behaviors, clinical care, social and economic factors, and the physical environment. While genetics and biology are recognized as predictors of health outcomes; they are neither modifiable nor measurable and so they are not included in the *Rankings* model. Each component comprises one or more subcomponents, which are defined by one or more measures from various data sources and assigned a weight based on its relative importance.

The process for determining weights for each of the components and for each of the measures within each component (see Table 1) was guided by several considerations, including a review of the literature around the impact of various factors on health outcomes, a historical perspective, weights used by other rankings, our own analysis of the variation of outcomes explained by each factor, and pragmatic issues involving communications and stakeholder engagement. Additional details regarding the choice of weights are available [27].

**Table 1:** **2014** ***County Health Rankings*** **: measures, data sources, and years of data**

	**Measure**	**Weight**	**Data source**	**Years of data**
**HEALTH OUTCOMES**				
Length of life	Premature death	50%	National Center for Health Statistics	2008-2010
Quality of life	Poor or fair health	10%	Behavioral Risk Factor Surveillance System	2006-2012
	Poor physical health days	10%	Behavioral Risk Factor Surveillance System	2006-2012
	Poor mental health days	10%	Behavioral Risk Factor Surveillance System	2006-2012
	Low birthweight	20%	National Center for Health Statistics	2005-2011
**HEALTH FACTORS**				
**Health Behaviors**				
Tobacco use	Adult smoking	10%	Behavioral Risk Factor Surveillance System	2006-2012
Diet and exercise	Adult obesity	5%	National Center for Chronic Disease Prevention and Health Promotion (NCCDPHP)	2010
	Food environment index^a^	2%	USDA Food Environment Atlas, Map the Meal Gap	2010-2011
	Physical inactivity	2%	NCCDPHP	2010
	Access to exercise opportunities^a^	1%	OneSource Global Business Browser, Delorme map data, Esri, & US Census Tigerline Files	2010 & 2012
Alcohol and drug use	Excessive drinking	2.5%	Behavioral Risk Factor Surveillance System	2006-2012
	Alcohol-impaired driving deaths	2.5%	Fatality Analysis Reporting System	2008-2012
Sexual activity	Sexually transmitted infections	2.5%	National Center for HIV/AIDS, Viral Hepatitis, STD, and TB prevention	2011
	Teen births	2.5%	National Center for Health Statistics	2005-2011
**Clinical Care**				
Access to care	Uninsured	5%	Small Area Health Insurance Estimates	2011
	Primary care physicians	3%	HRSA Area Resource File	2011
	Dentists	1%	HRSA Area Resource File	2012
	Mental health providers	1%	CMS, National Provider Identification	2013
Quality of care	Preventable hospital stays	5%	Medicare/Dartmouth Institute	2011
	Diabetic screening^a^	2.5%	Medicare/Dartmouth Institute	2011
	Mammography screening^a^	2.5%	Medicare/Dartmouth Institute	2011
**Social and Economic Factors**				
Education	High school graduation^a^	5%	data.gov, supplemented w/ National Center for Education Statistics	2010-2011
	Some college^a^	5%	American Community Survey	2008-2012
Employment	Unemployment	10%	Bureau of Labor Statistics	2012
Income	Children in poverty	10%	Small Area Income and Poverty Estimates	2012
Family and social support	Inadequate social support	2.5%	Behavioral Risk Factor Surveillance System	2005-2010
	Children in single-parent households	2.5%	American Community Survey	2008-2012
Community safety	Violent crime	2.5%	Uniform Crime Reporting - FBI	2009-2011
	Injury deaths	2.5%	CDC WONDER	2006-2010
**Physical Environment**				
Air and water quality	Air pollution - particulate matter^b^	2.5%	CDC WONDER	2011
	Drinking water violations	2.5%	Safe Drinking Water Information System	FY 2012 -2013
Housing and transit	Severe housing problems	2%	HUD, Comprehensive Housing Affordability Strategy	2006-2010
	Driving alone to work	2%	American Community Survey	2008-2012
	Long commute – driving alone	1%	American Community Survey	2008-2012

CDC WONDER: Centers for Disease Control and Prevention Wide-ranging Online Data for Epidemiologic Research; CMS: Centers for Medicare & Medicaid Services; ESRI: FBI: Federal Bureau of Investigation; HIV: human immunodeficiency virus; HRSA: Health Resources and Services Administration; HUD: Housing and Urban Development; STD: sexually transmitted disease; TB: tuberculosis; USDA: United States Department of Agriculture.

^a^Reverse coded measures.

^b^Not available for AK and HI.

### Geographical level of analysis

We use county as the geographical level of analysis for the *Rankings*. We include any entity that has its own Federal Information Processing Standard county code, which includes both counties and county equivalents. Examples of county equivalents include parishes in Louisiana; boroughs in Alaska; and certain major cities, such as Baltimore and St. Louis. Counties can vary widely in size; by both land area (e.g., San Bernadino County, CA = 20,000 square miles; Kalawao County, HI = 12 square miles) and population (e.g., Los Angeles County, CA = 9.9 million; Loving County, TX = 71). To facilitate inclusion of counties with smaller populations, we use two different strategies. We use the average of multiple years of data for several measures, giving equal weight to each observation year. This means that even small, sparsely populated places will have adequate numbers in terms of events or sample size for defining and reporting measures. Also, for places with insufficient sample size to report data (or missing values for other reasons) on any individual measure, we use the state mean as an estimate for that county. We chose this method in order to maintain the theme of being able to easily communicate our methods to the public. Of the 34 measures used in the 2014 *Rankings*, 12 measures have no missing data, six measures have 0%-1% missing, nine have 1%-10% missing, and seven have >10% missing (maximum 28% missing for binge drinking). These two strategies (multiple years of data and mean imputation) allow us to rank almost all of the 3,143 counties or county equivalents. In 2014, only 95 (3%) counties were unranked due to having one of the following: 1) a missing value for premature death, 2) an unreliable value for premature death with no other measure of morbidity available, or 3) an unreliable value for premature death and low birth weight with no other measure of morbidity available. Unreliable is defined as values for measures where the relative standard error was more than 20% of the estimated value—a threshold where estimates may be unreliable and should be interpreted with caution [28].

### Data sources and measures

The data for each of the components of the *Rankings* model are selected from a number of national data sources, including the National Center for Health Statistics, Behavioral Risk Factor Surveillance System, and American Community Survey, among others. A complete list of the data sources and measures used in the 2014 *Rankings* can be found in Table 1. The following criteria are applied to the selection of measures to represent each of the components of the model:

- ‣ Reflect important aspects of population health that can be improved
- ‣ Availability and reliability of indicators at the county level throughout the nation
- ‣ Ability for conditions underlying a measure to be modified through community action
- ‣ Valid, reliable, recognized, and used by others
- ‣ Available at low or no cost
- ‣ Recently and regularly updated
- ‣ Feedback from a panel of technical experts
- ‣ Alignment with America’s Health Rankings’ indicators
- ‣ Fewer measures are better than more

The most common reason we do not include a measure is lack of data, or of affordable data, at the county level. Also, because we wish to focus on the multiple factors that influence the overall health of counties, we do not include rates of specific diseases or their related risk factors. Similarly, we do not include measures of age or race/ethnicity in our calculation of ranks because the focus of the *Rankings* is on *modifiable* determinants of health, though all of the health outcomes measures except low birth weight are age-adjusted according to the 2000 US standard population.

### Calculating summary scores and ranks

Data for each of the measures are assembled, cleaned, and calculated by the *Rankings* staff, with two exceptions. Measures based on vital statistics data, sexually transmitted disease rates, and Behavioral Risk Factor Surveillance System survey data are calculated for the *Rankings* by staff at the National Center for Health Statistics and other units of the Centers for Disease Control and Prevention, and health care quality measures are calculated by the authors of the Dartmouth Atlas of Healthcare. Since the measures are based on different scales (percentages, rates, and averages of survey responses or other metrics), we standardize each measure within each state to the average of counties in that state. Standardizing each of these measures transforms them to the same metric, with a mean value of 0 and a standard deviation of 1. We refer to these as Z-scores where:

$$ \mathrm{Z}=\frac{\left( County\  Value\right)-\left( Average\  of\  Counties\  in\  State\right)}{\left( Standard\  Deviation\  of\  Counties\  in\  State\right)} $$

Each Z-score is relative to the other counties in that state (i.e., not compared to an absolute standard) and reported in the metric of standard deviations. A positive Z-score indicates a value higher than the average of counties in that state, and a negative Z-score indicates a value for that county lower than the average of counties in that state. For example, if a county has a Z-score on a measure of 1.2, that means the county is 1.2 standard deviations above the state average of counties for that measure. For counties with a population of 20,000 or less, we truncate any Z-score that is < −3.0 or > 3.0 to −3.0 or 3.0, respectively, to reduce the impact of outliers due to small area variations. For most of the measures, a higher Z-score score indicates poorer health (e.g., years of potential life lost before age 75). However, for some of our measures (e.g., high school graduation) a higher score indicates better health or a more desirable value. We take this into account before computing summary scores by multiplying them by −1, so that higher scores indicate poorer health.

After Z-scores are calculated, they are multiplied by their assigned weight and summed to create eight summary composite scores: overall health outcomes (including mortality and morbidity separately) and overall health factors (including health behaviors, clinical care, social and economic factors, and physical environment separately). Composite scores are then sorted from lowest to highest within each state. The lowest score (best health) gets a rank of #1 for that state and the highest score (worst health) gets whatever rank corresponds to the number of places we rank in that state.

## Results

**Figure 2:**
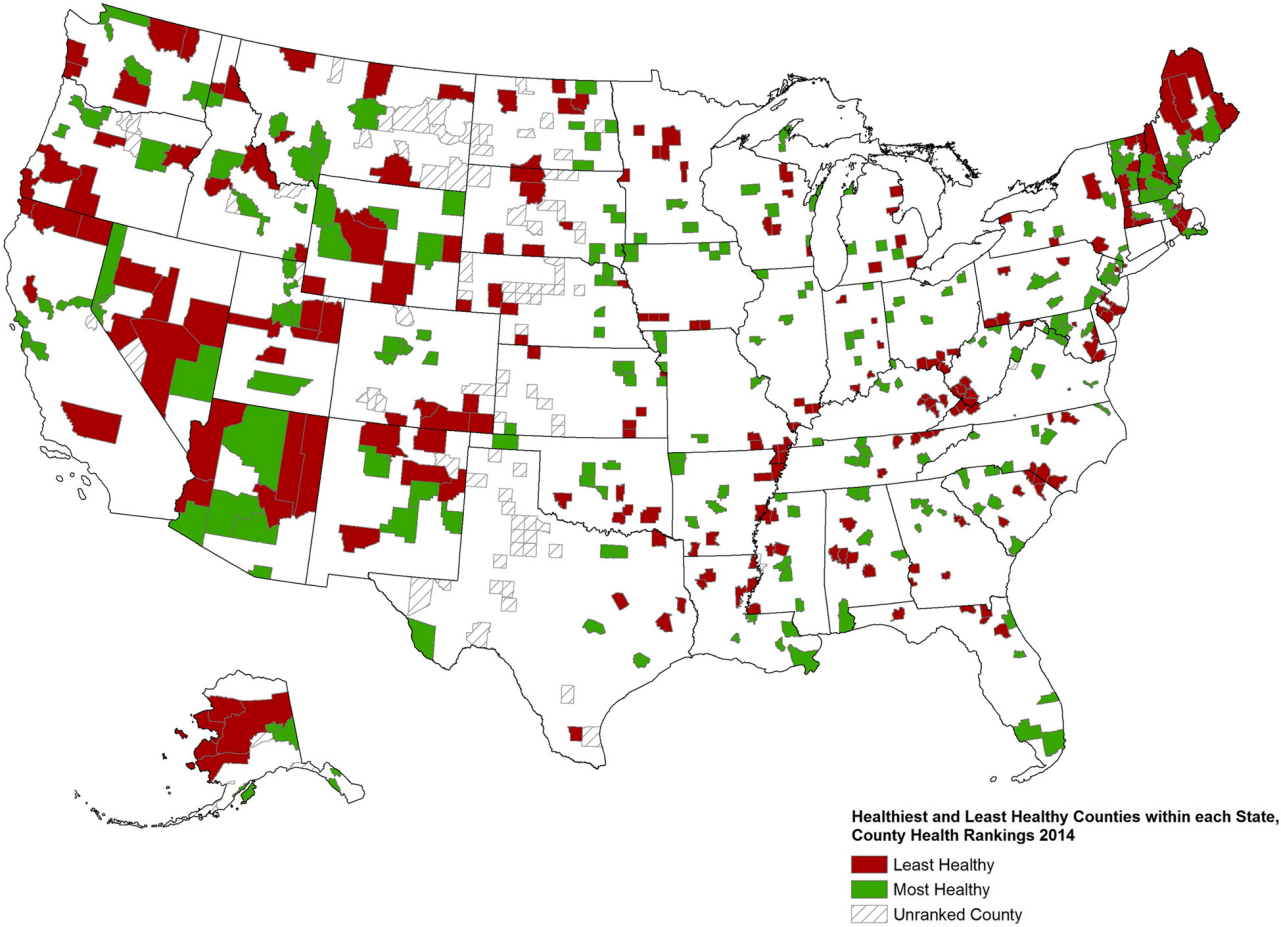
The five healthiest and least healthy counties within each state, based on the health outcomes ranking from the 2014 *County Health Rankings* (see: www.countyhealthrankings.org). Only states with at least 10 counties are shown. Hash marked counties reflect counties with insufficient data to rank.

The following includes a sample of 2014 *Rankings* findings to highlight several key points. Figure 2 shows the top five and bottom five counties within each state (with 10 or more counties) based on their within-state health outcome ranks. The map shows that in some states the healthiest and unhealthiest counties are located across the state, while in other states the healthiest and unhealthiest counties are adjacent to each other. Table 2 shows differences between the healthiest and unhealthiest counties (based on the health outcomes summary measure score) by individual measure. The five least healthy counties in each state have premature death rates that are more than twice the rates of the five healthiest counties. These counties with poorer health outcomes also have the highest rates of smoking, teen births, physical inactivity, preventable hospital stays, and children living in poverty.

**Table 2:** **Comparison of least healthy and healthiest counties by state for selected measurements, 2014 (Source: 2014** ***County Health Rankings*** **)**

	**All** ^**a**^	**Least healthy (LH)** ^**b**^	**Healthy (H)** ^**c**^	**Others** ^**d**^	**Ratio (LH/H)**
Number of counties	3,027	230	230	2,567	
**HEALTH OUTCOMES**					
Premature death (years of potential life lost under 75 yrs per 100,000 population)	7,746	10,862	5,113	7,785	2.1
Poor or fair health (%)	17%	20%	11%	17%	1.8
Poor physical healthy days (per month)	3.7	4.4	2.9	3.8	1.5
Poor mental health days (per month)	3.5	4	2.9	3.5	1.4
Low birth weight (% Live births <2500 g)	8%	9%	7%	8%	1.3
**HEALTH FACTORS**					
**Health Behaviors**					
Adult smoking (%)	21%	24%	16%	21%	1.5
Adult obesity (%)	31%	32%	27%	31%	1.2
Food environment (Index)	2.4	3.3	1.8	2.4	1.9
Physical inactivity (%)	28%	30%	23%	28%	1.3
Exercise environment (%)	54%	47%	69%	53%	0.7
Binge plus heavy drinking (%)	16%	16%	17%	16%	0.9
Alcohol-impaired driving deaths (%)	31%	31%	30%	32%	1.0
Chlamydia rate (per 100 k)	271	312	214	277	1.5
Teen birth rate (per 1,000 females aged 15–19)	42.4	55.43	21.61	43.53	2.6
**Clinical Care**					
Uninsured under age 65 (%)	18%	18%	15%	18%	1.2
Primary care physicians (population ratio)	1,980	2,087	1,472	2,003	1.4
Dentists (population ratio)	2,730	2,740	1,973	2,841	1.4
Mental health providers (population ratio)	1,640	1,379	1,131	1,728	1.2
Preventable hospital stays (per 1000 Medicare enrollees)	71	78	56	73	1.4
Diabetic screening (% of diabetic Medicare enrollees)	85%	83%	86%	85%	1.0
Mammography screening (%)	61%	57%	66%	61%	0.9
**Social and Economic Factors**					
High school graduation (%)	83%	77%	86%	83%	0.9
Some college (%)	55%	49%	68%	54%	0.7
Unemployment (%)	8%	9%	6%	8%	1.5
Children in poverty (%)	24%	31%	15%	24%	2.1
Inadequate social support (%)	19%	23%	17%	19%	1.4
Single-parent household (%)	31%	38%	24%	31%	1.6
Violent crime (per 100 k)	204.55	258.95	156.02	206.97	1.7
Injury mortality (per 100 K)	73	98.3	53.7	73.2	1.8
**Physical Environment**					
Air pollution – PM2.5 (micrograms per cubic meter)	11.93	11.8	11.5	11.98	1.0
Drinking water violations (%)	1%	3%	1%	1%	3.0
Severe housing problems (%)	14%	15%	14%	13%	1.1
Drives alone to work (%)	80%	78%	78%	80%	1.0
Drives their commute alone for more than 30 minutes (%)	29%	29%	30%	29%	1.0
**Additional Measures**					
2011 Population Estimate (mean)	26,837	18,300	75,755	26,310	0.2

^a^While there are 3,143 counties and county equivalents in the US, we did not include data for 116 of them. We lack data for 96 counties. The remaining counties were from states with less than 10 counties each (CT, DE, HI, and RI) that were excluded from this analysis.

^b^This column combines data for the five least healthy counties (based on the summary health outcome measure) in each state.

^c^This column combines data for five healthiest counties (based on the summary health outcome measure) in each state.

^d^This column includes all other counties not falling within the five least healthy or five healthiest counties in each state.

**Figure 3:**
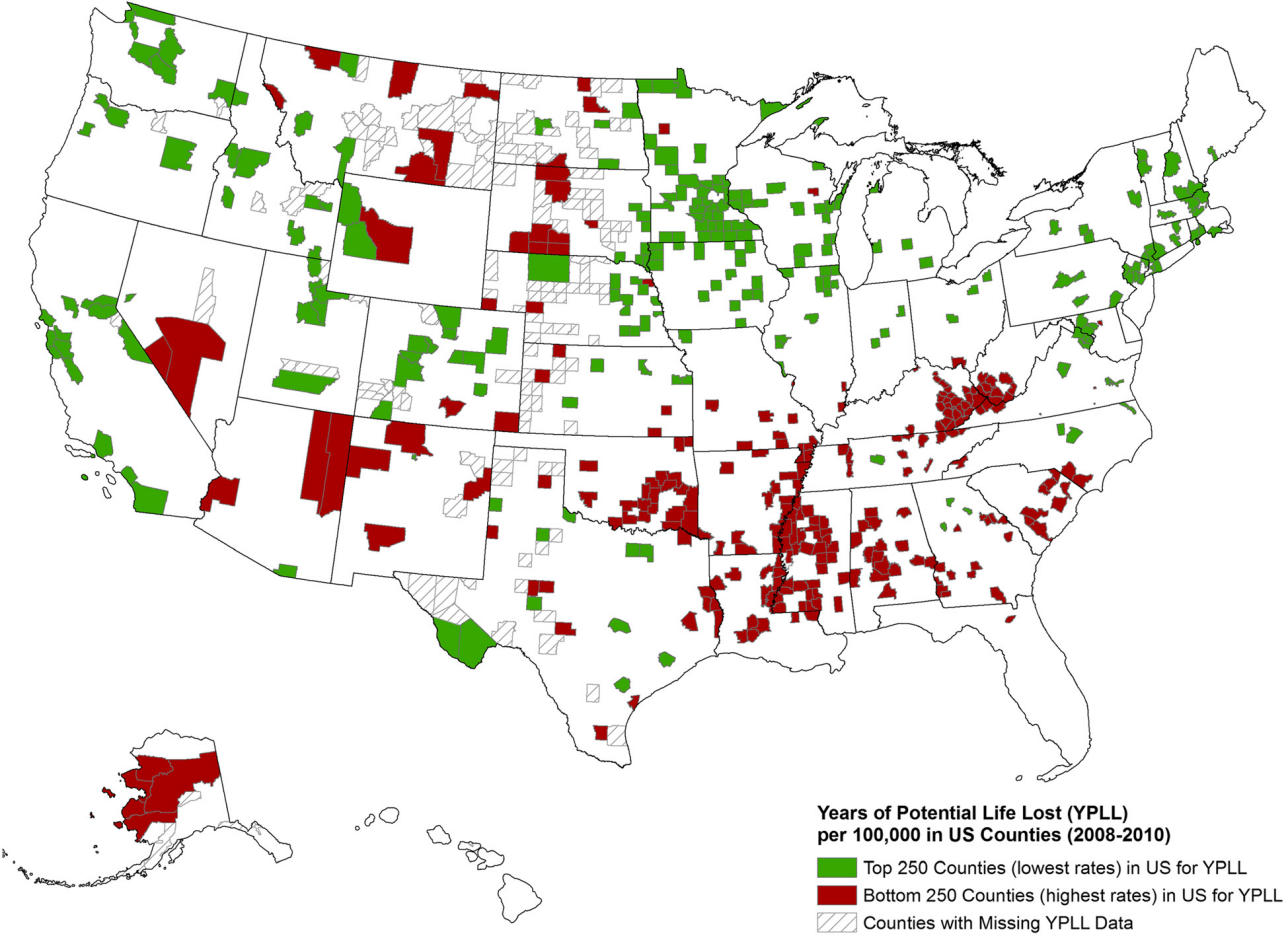
Counties with the lowest (green) and highest (red) premature mortality (years of potential life lost) rates in the nation, 2008–2010. Hash marked counties reflect counties with insufficient data to rank. Source: 2014 *County Health Rankings* (see: www.countyhealthrankings.org).

Figure 3 shows the 250 counties with the lowest premature death rates and the 250 counties with the highest premature death rates. Since premature death makes up 50% of the health outcomes composite score, this map shows a picture similar to what would be portrayed if we had chosen to rank counties nationally, rather than within-state. Rather than highlight the significant differences in health that exist within each state, this map shows distinct regional differences with clusters of high rates of premature death in the southern and Appalachian states and in the Plains states in counties where Indian reservations are located [29].

## Discussion

Using a model that summarizes the health outcomes and determinants of all counties throughout the nation, the *County Health Rankings* clearly demonstrates differences in health by place, raises awareness of the many factors that influence health, and stimulates community health improvement efforts. The use of ranking draws upon the human instinct to compete by facilitating comparisons between neighboring or peer counties within states. Ranking the health of places may also be controversial, leading to considerable interest in the media and among policymakers. This may explain the reluctance by governmental agencies to use rankings, and our experience that non-governmental foundations, including the Robert Wood Johnson Foundation and the United Health Foundation, support major health rankings in the United States.

The attention garnered by the *Rankings* has served as a catalyst toward action to promote health in communities across the country. Examples of the types of actions communities have been taken and different ways the data have been used are available at http://www.countyhealthrankings.org/roadmaps/stories and http://www.countyhealthrankings.org/roadmaps/projects. Also, our methodology has been adopted by public health organizations in other countries, for example the Asturias Health Rankings in Asturias, Spain [30].

### Strengths of the County Health Rankings approach

We believe that several characteristics of the *Rankings* contribute to its success. First, the model that we have developed is clear and easy to understand by the media and general public. The use of single summary composite measures of the current health (i.e., health outcomes) and factors that predict health outcomes in the future (i.e., health factors) of a county summarize underlying complex data to a form that policymakers and the general public can easily use. Also, the use of summary composite measures can have the effect of presenting the “big picture” in a way that is easier to interpret than trying to identify the trend in many separate measures [31]. This encourages users to “see the forest for the trees” and to not place undue emphasis on individual performance measures.

Second, by converting these summary composite scores into ranks, the *Rankings* have the potential to maximize public appeal and generate attention to the need for action. Although public health agencies at all levels publish many reports each year, they often go unnoticed by the media and general public. By ranking health outcomes and health factors, we can increase awareness of health information that might otherwise go unnoticed. Rankings serve as a hook for the media and play on our competitive instincts. It is much easier to say “the best” or “the worst” than it is to compare relative or absolute differences. This experience is not unique to health rankings and has been noted in rankings of health care organizations and educational institutions. Hazelkorn [32] suggests that the practice of “naming and shaming” has introduced a competitive element that has positively influenced institutional behavior and increased the quality of higher education.

It is important to note that the *Rankings*, reported annually at www.countyhealthrankings.org, rank counties within states rather than across state borders or across the nation (e.g., as shown in Figure 3). This approach is necessary as a number of the measures are not comparable across states because the models used to construct them emphasize state rates and are, therefore, state specific. More importantly, not providing national rankings compels counties to compare the health of where they live to their neighboring counties or other counties in their state. We believe this is more likely to inspire change and is more in line with the overall goal of the *Rankings* – to mobilize action toward community health. For instance, the knowledge that a county is ranked last in their state for an outcome should be more informative and motivating to take action than the knowledge of being ranked 500^th^ or 2,500^th^ in the nation. However, we do provide a national benchmark representing the best 10% for each measure so that counties can have a sense of how their health outcomes or factors compare to the top counties in the nation. In addition, we direct users of the Rankings to other resources, such as the *Community Health Status Indicators Project* (available at: http://wwwn.cdc.gov/CommunityHealth/homepage.aspx), which permit counties to compare their health indicators with peer counties across the nation [33].

### Limitations of the County Health Rankings

Despite these advantages, several limitations exist in the *Rankings* and in health rankings in general. First, it is true of all health rankings that there is no perfect model to summarize the health of an entire population [34]. Value judgments are inherent in selecting measures to be included in the model, as well as the weights used for combining these measures into a summary index. A recent study by Hendryx et al. [35] examined the correlations between the 4 categories of health factors with the health outcomes for the 2012 *Rankings* and found that the measures of social and economic conditions and health behaviors were more closely related to the health outcomes measure than measures of health care and the environment. We chose not to adjust health behavior measures by social and economic factors, since the crude measures better reflect actual burden in the county, and interventions may be designed to address both behaviors directly and their underlying social and economic determinants.

Second, rankings are on an ordinal scale, which means close ranks are not necessarily statistically significantly different from each other. For example in the *Rankings*, the top-ranked county in a state (#1) is not necessarily significantly healthier than the second-ranked county (#2) [3]. To complement the individual county ranks, we group counties into quartiles according to their health outcomes and health factors ranks and provide color-coded quartile maps for each state of ranks to facilitate understanding of the distribution of health within each state. Lastly, we also acknowledge that ranks may not be useful for measuring changes over time. Assuming the same measures are used each year, improvements in a county’s rank from year to year may be due to real improvement in health in that county or could possibly be due to declines in health in other counties. Our decision to update the *Rankings* annually likely exacerbates this issue, but we believe this is offset by the benefits of continuing to maintain momentum around community health improvement.

Another concern involves the reliability of estimates, particularly for counties with smaller populations; we recognize that the reliability of our measures does vary. Mortality data, which are reported almost 100% of the time, are extremely reliable as counts of death, while other measures (e.g., excessive drinking) are missing for many counties, and still other measures (e.g., air quality and obesity rates) are based on modeling methods. A sensitivity analysis showed that using different strategies for missing/unreliable data and outlier values led to only small changes in rank when compared to the reference model [36]. We try to provide supporting information to help users understand the quality of those measures. For example, within each of our county snapshots we provide the margin of errors (95% confidence intervals) for the data that comprise our indicators. We also make it clear that data from the *Rankings* should be used as a starting point, not an end point, and we encourage users to look to local sources of data to understand more about the health of their community. For example, in New England counties do not necessarily reflect the structure of local government. In large urban counties such as Los Angeles County, county-level statistics may not be especially useful, whereas in sparsely populated areas, counties are too small as units of analysis since many services are delivered by groups of counties. Finally, combining data from low-income urban neighborhoods with wealthier suburbs in the same metropolitan county masks these health disparities.

### Future opportunities for the *County Health Rankings*

Several opportunities exist that could improve the quality and usefulness of the *County Health Rankings*. Future efforts could build upon our model and experience, to go beyond within-state county rankings, to draw attention to health differences across state boundaries (e.g., regional rankings) or within counties, such as large metropolitan areas. Many communities have already started to “connect the dots” by linking individual- and community-wide data to produce estimates of health outcomes and health factors at subcounty and even census track levels [37]. Future efforts could leverage the publicity of *County Health Rankings* to guide media and stakeholder groups to further explore place-based disparities at a more micro level. For example, Denver Health uses electronic health record data to track obesity (and other risk factors) on a census tract level [38] and Public Health – Seattle & King County in Washington State is using small area estimation to rank census tracts on health and social well-being measures [39].

Since the measures with the highest percent missing come from the Behavioral Risk Factor Surveillance System, we are working with the Centers for Disease Control and Prevention to obtain modeled estimates for these measures. In addition, as more years of data are available, analyses of trends in health outcomes or a number of the health factors will be possible—especially for those measures that are reported using single-year or up to three-year averages. Future analyses could be done to compare those counties that have improved in rank with their states compared to counties whose rank has declined. These analyses must account for variance in rank estimates to assure that changes are due to real differences in trends, rather than variation in the underlying data. With funding from the Robert Wood Johnson Foundation, the University of Wisconsin Population Health Institute will be providing small grants to organizations interested in pursuing these and other potential studies.

## Conclusions

In conclusion, the *County Health Rankings* can be used to clearly demonstrate differences in health by place, raise awareness of the many factors that influence health, and stimulate community health improvement efforts. The *Rankings* draws upon the human instinct to compete by facilitating comparisons between neighboring or peer counties within states. Since no population health model, or rankings based off such models, will ever perfectly describe the health of its population, we encourage users to look to local sources of data to understand more about the health of their community.
